# Progress in Surgery of the Liver and Pancreas

**Published:** 1901-08-24

**Authors:** 


### Progress in Surgery of the Liver and Pancreas.

**Cirrhosis of the Liver.**—This, when treated by suturing the liver and omentum to the abdominal parietes should, according to Frazier,[1] be operated upon under local anæsthesia, as the individuals are usually alcoholics and belong to a class in which ether narcosis of itself has a very material effect upon the mortality. This operation purports to open another channel for the relief of the obstructed portal circulation, though normally there exists a more or less free collateral circulation between the systems of the portal vein and the inferior vena cava. Thus the coronaries anastomose, through the œsophageal plexus, with the azygos veins; the veins of the cæcum and colon with the internal mammary; the hypogastric with the hæmorrhoidal; the veins of the hepatic ligament with those in Glisson's capsules; and the veins of the round ligament with the epigastric. The author thinks that in properly selected cases this mode of treatment is promising, and he would limit its application to: 1. Cases in which the liver is cirrhotic. 2. Cases in which there is reason to believe the liver cells are not devoid of function. 3. Cases in which internal medication (particularly iodide of potassium) and paracentesis fail to afford relief—or, in other words, in utterly hopeless cases—and 4. Cases in which there is no reasonable contra-indication. Jelks,[2] quoting Frazier's statistics, gives the mortality as 21 per cent., unimproved 28 per cent., improved 8 per cent., and recovery 43 per cent.

**Resection of the Liver.**—Segale[3] believes that failures, attended by a fatal primary or secondary hæmorrhage, are especially dependent on the insufficiency of the methods of hæmostasis now in use, most sutures taking as their *point d'appui* the hepatic and peri-hepatic tissues which have a very feeble resistance. No account is taken of the compressibility of the liver substance; thus it happens that even when the suture is effective at the time of operation it afterwards ceases to be so, with the result that secondary hæmorrhage occurs. Segale has therefore devised a method after the fashion of the quilled suture, but in place of quills he uses small beads of ebony or ivory; these beads, which are hollowed in the middle, fit into each other at the ends, and are threaded together with catgut so as form a small rod. The material was chosen because it lends itself to disinfection, while the bead-like arrangement favours the natural encapsulation of the component parts of the rod which are incapable of absorption. The mode of use is as follows:—At the boundary of the lobe of the liver which it is intended to remove, and along a transverse line, there is passed a series of loops of elastic thread of sufficient resistance for the loops to stick out on the lower surface of the liver; one of the little rods is passed through these loops by which it is held fixed, while the other is placed on the upper surface between the two free ends of the thread; these are next drawn sufficiently tight and fixed with knots of catgut. The resection is then carried out, care being taken to make the two incisions in such a fashion as to have a solution of continuity triangular in section, with the apex directed towards the middle; in this manner it is possible to bring together the free surfaces by suture. The rods become broken up after the absorption of the catgut and the small pieces are easily encapsuled.

**Echinococcus Cyst of the Liver.**—This is usually treated, says Fowler,[4] by stitching the sac wall to the edges of the wound in the abdominal parietes, and then incising it, either in one or two stages. Subsequent to incision, the lining membrane of the sac is peeled off as completely as possible. The cavity is irrigated and packed daily until only a small sinus is left. Because of the long period during which the cases drained are kept from active work, it is desirable in those cases which admit of the procedure being carried out with a fair prospect of success, that a hepatectomy be performed in order to totally eradicate the disease. Cysts which are not very large, or where a comparatively small amount of liver tissue is involved, may be subjected to this procedure. Great care must be exercised in selecting such cases. Loux[5] reports a case of resection of the liver for echinococcus cyst, carried out by means of the paquelin cautery; and Smith and Macready[6] relate the successful drainage of a case of hydatids of the liver occurring after an interval of ten years in the pleural and peritoneal cavities.

**Abscess of the Liver.**—Smits[6] says that the classical indications, both objective and subjective, of liver abscess must be regarded as unreliable, and usually occur in an advanced stage of the disease, when the prospects of operative treatment have become unfavourable. Much value is attached to exploratory puncture as a means of diagnosis. The author in discussing the objections to exploratory puncture, especially those made on the probable dangers of septic infection and hæmorrhage, states that when pus is found a radical operation should at once be practised, and that the needle should not be used when the abscess is supposed to be seated near the anterior margin of the liver or in the left lobe. In such cases he would substitute a free exposure of the liver by exploratory laparotomy. Exploratory puncture, when practised with strict regard to asepticity, is regarded as an absolutely safe procedure which, the author asserts, it is the duty of the surgeon to practise whenever there is the possibility of the existence of an abscess in the liver, and to repeat until he has found the purulent collection, or has been convinced by the results of such investigation that no pus is present. Three cases of successful operation for liver abscess are reported by Dick,[7] and Daniel[8] has successfully treated a case of supra-hepatic abscess by the trans-thoracic route. Swain[9] gives a summary of our knowledge of diseases of the liver, gall-bladder, and biliary ducts.

**Contusions of the Liver.**—Gosset[10] reports two cases of wound of the liver caused by a kick from a horse, and treated by laparotomy. The author draws attention to the value of contraction of the abdominal wall in contusions of the abdomen. He says he has never omitted, whenever this has been present, to perform laparotomy, and he has always found visceral lesions present; abstention in its absence has also been justified. The patient must be quietened and made to breathe with the mouth open; then one must place the hand on the flat very gently and press it down slowly and progressively. When there is no lesion one can press as deeply as one wishes. In case of very marked contraction there is no difficulty, as then the whole abdominal wall is like a board.

**Pancreatitis.**—This is dealt with by Robson.[11] The essential and immediate cause of the various forms of this disease is bacterial infection, but as in inflammatory affections of the liver and bile ducts we look for extrinsic causes, so in pancreatic diseases we find biliary and pancreatic lithiasis, injury, gastro-duodenal catarrh, ulcer and cancer of the stomach, pylorus or duodenum, such as typhoid fever and influenza, to be determining factors, though in some cases pancreatitis has come on suddenly in persons in robust health, and the determining cause has been beyond recognition. In acute infective pancreatitis treatment practically resolves itself into that of peritonitis commencing in the superior abdominal region. The symptoms are apt to resemble those of intestinal obstruction. Just as in a perforative or gangrenous appendicitis an early evacuation of the septic matter is necessary to recovery, so in this equally lethal affection an early exploration from the front through the middle line above the umbilicus, or from behind through the left costo-vertebral angle is demanded in order to evacuate the septic material and adopt free drainage. The subacute form of pancreatitis is more amenable to treatment, as the indications are so much more definite and there is more time for careful consideration; and though it has usually only been attacked when an abscess was formed, and is manifestly making its way to the surface, yet there is no reason why in some cases surgical treatment should not be adopted at an earlier stage. Chronic pancreatitis must also be treated by abdominal section and drainage, but in this case the drainage is indirect and obtained by draining the gall-bladder by cholecystotomy, cholecystenterostomy, or duodeno-choledochotomy, but the exact line of treatment cannot be determined until the abdomen is opened. Bailing[12] refers to some cases of chronic enlargement of the pancreas in association with or producing attacks simulating biliary colic. Richardson[13] thinks that in all obscure inflammations of the epigastrium, attended by acute sudden pain, we ought to explore. In all of the cases which the author has seen in which the diagnosis of acute pancreatitis was confirmed, death has followed.

[1] Amer. Jour. Med. Sci., Dec. 1900, p. 661. [2] Med. Rec., March 23, 1901, p. 455. [3] Brit. Med. Jour., Oct. 6, 1900, Epitome No. 142. [4] Annals of Surgery, Dec. 1900, p. 821. [5] Ibidem, Nov. 1900, p. 714. [6] Brit. Med. Jour., Feb. 2, 1901, Epitome No. 78. [7] Ibidem, March 9, 1901, p. 576. [8] Lancet, April 6, 1901, p. 1015. [9] Practitioner, Nov. 1900, p. 535. [10] Quarterly Med. Jour., Nov. 1900, p. 61. [11] Brit. Med Jour., May 11, 1900, p. 1129 [12] Ibidem, Dec. 22, 1900, p. 1768. [13] Med. Rec., March 16, 1901, p. 425.

